# Multi‐omics identifies promoter methylation and gene expression changes associated with human skeletal muscle atrophy

**DOI:** 10.1113/EP093999

**Published:** 2026-08-24

**Authors:** Jamie‐Lee M. Thompson, Thomas M. Doering, Boris P. Budiono, Kristen L. Mackenzie‐Shalders, Kevin J. Ashton, Paul J. Dunn, Vernon G. Coffey

**Affiliations:** ^1^ Bond Institute of Health and Sport and Faculty of Health Sciences and Medicine Bond University Gold Coast Queensland Australia; ^2^ Victor Chang Cardiac Research Institute, Darlinghurst Sydney Australia; ^3^ University of New South Wales Sydney New South Wales Australia; ^4^ School of Health Medical and Applied Sciences Central Queensland University Rockhampton Queensland Australia; ^5^ College of Health and Enablement Flinders University Adelaide South Australia Australia

**Keywords:** genetics, unloading, wasting

## Abstract

Skeletal muscle atrophy is a secondary complication in the aetiology of injury and chronic disease. Identifying mechanisms that control muscle mass is necessary to characterise atrophy and develop prevention strategies. We aimed to integrate transcriptomic and epigenomic data to identify key regulatory pathways controlled by promoter DNA methylation during muscle unloading. Twenty‐one healthy men (20–40 years) completed a 4‐week standardised exercise programme prior to a 14‐day knee brace immobilisation with dietary control. Skeletal muscle mass and strength were assessed before and after immobilisation and biopsies were collected (m. vastus lateralis) before, at 3 days, and at completion at 14 days. RNA and DNA were isolated and analysed using Illumina RNA sequencing and DNA methylation 850K EPIC BeadChips. The 14‐day immobilisation decreased muscle mass (∼9%; *P *< 0.0001) and strength (∼16%; *P *< 0.0001). At 3 days, most biological processes (BPs) were upregulated/hypomethylated (157 gene sets); upregulated BPs included cell signalling and protein ubiquitination and downregulated BPs included metabolism. After 14 days, BPs were predominantly downregulated/hypermethylated, including translation and ribosome biogenesis. Across both time points, *HDAC4*, *GADD45A* and *CHRNA1* emerged as methylation‐regulated candidate mediators of atrophy. *HDAC4* and *GADD45A* showed strong correlations primarily at day 3, and *CHRNA1* remained significant at both time points, extending prior observations in animals to human skeletal muscle. We have characterised changes in gene expression related to hypo‐ and hyper‐methylation during muscle unloading in humans. These data extend our understanding of the regulatory processes that occur during skeletal muscle atrophy that, at the individual gene level, may be useful in developing strategies for reducing muscle wasting.

## Abstract (highlights)

- **What is the central question of this study?**
  What are the characteristics of global gene expression in skeletal muscle in response to limb immobilisation in young men?
- **What is the main finding and its importance?**
  Data obtained support the role of DNA methylation as a regulator of transcription and that methylation appears to impair gene expression for protein synthesis early and later amplify protein breakdown over 14 days of immobilisation. There are associations between methylation and transcription within biological processes as putative molecular targets to mitigate the debilitating impacts of muscle wasting.

## 1 INTRODUCTION

Skeletal muscle atrophy occurs in many different contexts such as bed rest (disuse), ageing (sarcopenia), malnutrition, cachexia and denervation (Cohen et al., 2015). Injury is also a common reason for limb immobilisation, and the associated non‐weight‐bearing and extended periods of recovery required during treatment will often result in a loss of 5–8% of muscle mass during short‐term limb immobilisation (Wall & Van Loon, 2013). Muscle mass is tightly regulated by interactions between genetic, environmental and behavioural factors. Previous studies have focused on characterising the molecular responses during protein degradation and regulation of the ubiquitin–proteasome system (UPS), and altered protein synthesis through processes regulating the mechanistic target of rapamycin (mTOR)/Akt pathway (Yoon, 2017). Together, these pathways are the most well‐characterised mechanisms associated with control of muscle mass, but the precise mechanisms regulating skeletal muscle atrophy remain unclear.

Most molecular and cellular candidates associated with skeletal muscle atrophy appear to be related to downregulation of mitochondrial and other metabolic pathways in skeletal muscle, including carbohydrate metabolism, fat oxidation, the citric acid cycle, electron transport chain, oxidative phosphorylation, and mitochondrial biogenesis (Abadi et al., 2009; Doering et al., 2022; Koopman et al., 2014; Powers & Jackson, 2008). Other notable downregulated biological processes associated with loss of muscle mass in humans include cell cycle regulation, cell growth and stress response (Chen et al., 2007). The role of DNA methylation as an epigenetic regulator of muscle atrophy has received less attention, but hypomethylated candidate genes *Ubr5*, *Trim63*, *Fbxo32* and *Chrna1* have been identified after 3 days and 14 days of atrophy in rats (Fisher et al., 2017; Seaborne et al., 2019). In humans, increased *PPARGC1A* DNA methylation has been observed following 10 days of bed rest, and methylation levels did not return to baseline after 4 weeks of aerobic exercise training (Alibegovic et al., 2010). In addition, a sarcopenia study in women aged 65–80 employing EPIC methylation arrays identified methylation changes in pathways such as muscle function, actin cytoskeleton organisation and energy metabolism (He et al., 2019). Accordingly, it seems plausible that DNA methylation is an important regulator of muscle atrophy.

Individual omic datasets from the transcriptome and methylome provide novel data, but the advent of multi‐omic tools now provides the ability to integrate these datasets to provide new information on the interactions between different regulatory systems. Therefore, integrative analysis of multi‐omics data can provide new understanding of the relationship between promoter methylation and mRNA expression at the omic scale. While prior work has begun to examine concurrent methylome and transcriptome changes in human skeletal muscle (Blocquiaux et al., 2021), these studies have been constrained by small sample sizes (*n* = 5–6) and were not specifically designed to characterise the early time course of immobilisation‐induced atrophy. The present study is, to our knowledge, the first to perform a fully integrated, paired analysis of the promoter methylome and transcriptome at defined time points during progressive limb immobilisation in humans, with a highly controlled study design and sample size (*n* = 21) that affords a worthwhile characterisation of the regulatory landscape of muscle atrophy. Accordingly, the aim of this study was to utilise multi‐omic interrogation of transcriptome and methylation data to identify key regulatory pathways associated with human skeletal muscle atrophy during unloading.

## 2 METHODS

### 2.1 Ethical approval

Prior to commencing the study each participant underwent an initial assessment with a member of the research team to determine their suitability for the study and to provide their written informed consent. Ethical approval for the study was obtained from the Bond University Human Research Ethics Committee (HREC reference: 0000015478) and conformed to the standards set by the *Declaration of Helsinki*. The study was prospectively registered as a clinical trial with the Australian New Zealand Clinical Trials Registry (ACTRN12616001399482).

### 2.2 Participants

Twenty‐one healthy men aged 20–40 years volunteered to participate in this study (mean age 26.0 ± 5.7 years, body mass 81.0 ± 11.6 kg, height 179.5 ± 6.8 cm, peak oxygen uptake (${\dot{V}}_{O_{2}\mathrm{peak}}$) 42.8 ± 6.9 mL kg min^−1^, unilateral leg press 3 repetition maximum (3RM) strength 130.4 ± 26.0 kg). The inclusion criteria were that the participants were between 20 and 40 years old and had been regularly exercising (3–5 times per week) for at least 6 months leading up to their commencement in the study.

### 2.3 Study design

The study was undertaken over an ∼8 week period and included (i) preliminary testing for exercise prescription, (ii) a 28 day standardised exercise training period, (iii) a 21 day dietary control period beginning 7 days before knee brace immobilisation, (iv) pre‐immobilisation testing of anthropometry and muscular strength, (v) 14 days’ knee‐brace leg immobilisation with skeletal muscle biopsies obtained on day 0, 3 and 14, and (vi) post‐immobilisation testing of anthropometry, ${\dot{V}}_{O_{2}\mathrm{peak}}$ and muscular strength.

The study was undertaken with men to minimise biological variability in epigenomic and transcriptomic outcomes. Emerging evidence from animal models suggests that transcriptomic profiles during the onset of disuse‐induced atrophy may diverge substantially between sexes, with distinct patterns of proteasomal, mitochondrial and myogenic gene regulation observed in male and female mice (Tsitkanou et al., 2023). Given that sex‐based differences in baseline DNA methylation have been shown in skeletal muscle and may modulate the epigenetic response to unloading, restricting this initial characterisation to male participants was considered necessary to establish a controlled reference dataset (Landen et al., 2021).

### 2.4 Standardisation of exercise training and dietary control

The 4‐week standardised exercise period prior to knee brace immobilisation was employed to normalise the relative training stimulus for all participants before inducing muscle atrophy. Baseline testing for the exercise prescription included unilateral three repetition maximum (3RM) testing of muscular strength, and a bilateral incremental cycling test to volitional fatigue to establish an estimate of peak oxygen uptake (${\dot{V}}_{O_{2}\mathrm{peak}}$). There was no difference in 3RM leg strength, which was closely matched between limbs (group mean: right 130.7 ± 25.6 kg versus left 130.4 ± 26.0 kg). Diet control was for 3 weeks’ duration, commencing 1 week before immobilisation and continuing until the last day of immobilisation. A commercial meal delivery system (direct to home) was contracted (24% protein; Light n’ Easy, Brisbane, Australia). Details of the physiological testing, 4‐week standardised training programme and dietary control have been previously described (Doering et al., 2022).

### 2.5 Limb immobilisation, MRI and DXA scans

A commercial leg brace (X‐ACT DonJoy Orthopedics, Lewisville, TX, USA) was fitted to the left leg and fully locked (60° knee flexion) preventing movement over the 2‐week immobilisation period. Unique cable ties were also fitted to ensure the brace could not be removed without being detected. Forearm crutches were provided to enable participant ambulation during the immobilisation period without weight‐bearing on the immobilised leg.

The knee brace immobilisation model employed in this study closely approximates the period of restricted limb loading that commonly follows musculoskeletal injury (including ligament reconstruction, fracture management, and orthopaedic surgery) where enforced non‐weight‐bearing and immobilisation for several weeks is standard clinical practice (Wall & Van Loon, 2013). Understanding the molecular mechanisms underpinning atrophy in this context is therefore directly translatable to improving recovery strategies in clinical and sports medicine settings.

Briefly, participants underwent DXA scanning (Lunar Prodigy, GE Medical Systems Lunar, Madison, WI, USA) and MRI (3T Magnetom Skyra, Siemens Healthineers, Forchheim, Germany) following an overnight fast. Vastus muscle group and quadriceps femoris cross‐sectional area (CSA; cm^2^) was measured on the middle scan by manual tracing (OsiriX Lite 8.0, Pixmeo, Bernex, Switzerland) (Rosset et al., 2004). Additional details on scanning methodology for the present study have been published previously (Doering et al., 2022).

### 2.6 Sample collection and RNA extraction

Muscle biopsies were taken in the early morning after an overnight fast from the left leg vastus lateralis with the biopsy site located midway between the superior border of the patella and the inguinal fold on the lateral aspect of the leg. Biopsies were taken via a separate incision from distal to proximal. Immediately after removal, the muscle tissue was separated from any visible connective tissue and fat and rinsed with 60% ice‐cold NaCl solution to remove excess blood. The sample was then snap‐frozen in liquid nitrogen and placed into long‐term storage at −80°C. The initial (day 0) biopsy was taken immediately before the participant was fitted with the leg brace; the day 3 biopsy was obtained supine on a plinth with the brace removed and then immediately refitted; the final (day 14) biopsy was taken immediately after the leg brace was removed.

### 2.7 RNA extraction and sequencing

Total RNA was isolated from 63 muscle biopsies (21 individuals, 3 time points; 0, 3 and 14 days) using the AllPrep DNA/RNA/miRNA Universal kit (Qiagen, Hilden, Germany) according to manufacturer's instructions. RNA sequencing was performed by the Australian Translational Genomics Centre located at Queensland University of Technology/Translational Research Institute. The TruSeq Stranded Total RNA Library kit with Ribo‐Zero Gold rRNA depletion (Illumina, San Diego, CA, USA) was used for library construction. Samples were pooled and sequenced on a NextSeq 500 system (Illumina) across eight flow cells, generating approximately 62 million paired‐end 2 × 75 bp reads per sample.

### 2.8 Differential gene expression analysis

Quality control of the raw FASTQ files was performed using the FastQC software package version 0.11.7. Transcript quantification was performed by quasi‐mapping to the human reference transcriptome (cDNA and ncRNA; Ensembl GRCh38 release 91) using Salmon version 0.9.1 (Patro et al., 2017). Transcript level estimates were imported into R/Bioconductor and summarised at the gene level using the tximport package version 1.18.0 (Soneson et al., 2016). Genes with a median count per million of <0.5 across all 63 samples were excluded from the analysis. Trimmed mean of *M*‐values (TMM) normalisation was used to correct for composition bias and library size before voom transformation and descriptive statistics. Differential expression analysis using linear modelling and empirical Bayes methods was performed in limma version 3.46.0 (Conesa & Nueda, 2017; Ritchie et al., 2015). Paired sample comparisons were employed using participant ID as a blocking factor in the design matrix, to compare immobilised (day 3 or day 14) and baseline (day 0) states within participants. The linear model also incorporated RIN as a covariate (mean RIN: 8.2; range: 7.1–9.3). A false discovery rate (FDR; Benjamini–Hochberg) was applied to correct for multiple testing, with statistical significance accepted at FDR < 0.05.

### 2.9 DNA extraction and methylation arrays

Genomic DNA was co‐extracted with total RNA. The DNA methylation investigation was performed by the Australian Translational Genomics Centre located at Queensland University of Technology/Translational Research Institute. Each DNA sample underwent bisulfite conversion using the EZ DNA Methylation Kit (Zymo Research, Irvine, CA, USA). Genome‐wide DNA methylation patterns in skeletal muscle were assessed using Infinium Methylation EPIC BeadChips (Illumina).

### 2.10 Differential methylation analysis

Channel intensities in IDAT files were analysed using minfi 1.28.0. The distribution of methylation values was assessed using a beta density plot to check for probe type bias. Filtering was performed based on *P*‐value detection (>0.01), beadcount (<3 in at least 5% samples), non‐CpG probes, cross‐reactive probes (McCartney et al., 2016; Pidsley et al., 2016), and probes containing single nucleotide polymorphisms (SNPs; Zhou et al., 2017). The beta‐mixture quantile (BMIQ) method was used to normalise the data (Teschendorff et al., 2013). Principal component analysis (PCA) was employed to assess covariates driving variation in the methylation data using the PCAtools package (Blighe, K. & Lun, A., 2021). The RUVm (Removing Unwanted Variation for differential methylation) approach in the missMethyl package was used to adjust data for bias, including batch and slide effects (Maksimovic et al., 2015). Probes were annotated with Illumina methylation EPIC annotation (Hansen, 2016). Promoter methylation of a differentially methylated gene (DMG) was defined by mCSEA as a minimum of five differentially methylated probes within or in close proximity (1500 bp upstream of the transcriptional start site (TSS1500) down to and including the first exon) to a gene promoter (Martorell‐Marugán et al., 2019).

### 2.11 Multi‐omic analysis and data integration

The mCSEA package (Martorell‐Marugán et al., 2019) was used to integrate RNA sequencing and DNA methylation data. This method is used to detect moderate, consistent and coordinated changes in DNA methylation patterns to identify promoter DMGs. Genes present in both the RNA‐seq and DNA methylation datasets were first identified and then integrated using the mCSEAIntegrate function. Pearson's correlation coefficient was then calculated between the paired methylation value and gene expression for any genes located 1500 bp up‐ or downstream of the methylation region. Negative correlations were considered significant at FDR < 0.1 for genes showing promoter hypomethylation and increased gene expression, or vice versa. Gene Ontology (GO) over‐representation analysis (ORA) was used to identify enriched GO biological processes (BPs) for the significant (FDR < 0.1) hypomethylated/upregulated genes and hypermethylated/downregulated genes separately, from each time point comparison (day 3 vs. day 0 and day 14 vs. day 0). These specified lists of genes were assessed for over‐representation against GO‐BP gene sets using clusterProfiler (Yu et al., 2012). A moderately permissive threshold (FDR < 0.1, nominal *P* < 0.01), with the gene set size range set to 25–500, was used at the correlation step to accommodate the greater heterogeneity of paired multi‐omic data with false positives controlled downstream by requiring a consistent negative correlation direction and pathway‐level convergence.

Significant pathways for each time point comparison were imported into Cytoscape using the EnrichmentMap package (Shannon et al., 2003). Pathways were clustered using the AutoAnnotate package (Kucera et al., 2016). Genes within clusters of interest were further visualised in Cytoscape using the geneMANIA package (Warde‐Farley et al., 2010). Interactions were based on co‐expression and the top 20 genes with the highest co‐expression outside of the cluster of interest were included. In addition, genes not expressed within our RNA‐seq dataset were excluded.

### 2.12 Statistical analysis

Pre‐ and post‐immobilisation data for 3RM strength, ${\dot{V}}_{O_{2}\mathrm{peak}}$ and skeletal muscle CSA were analysed using a two‐tailed paired Student's *t*‐test. All physiological and genomic data are expressed as means ± standard deviation (SD) and significance was accepted when *P *< 0.05 unless stated otherwise (GraphPad Prism 10, GraphPad Software, Boston, MA, USA). Given emerging studies on the effects of unloading on neuromuscular junction transmission in skeletal muscle (Sarto et al., 2025, 2022), Pearson correlations were undertaken in R to determine associations between prominent individual expression of genes implicated in formation, maintenance or transmission at the neuromuscular junction and loss of muscle strength (Li et al., 2018; Tintignac et al., 2015). A whole‐transcriptome screen and leave‐one‐out checks confirmed the main results were not attributable to any single participant.

## 3 RESULTS

### 3.1 Phenotype characteristics

There was a significant decrease in vastus group and quadriceps femoris CSA, and total leg lean mass, after 14 days immobilisation (Doering et al., 2022). Specifically, the combined vastus medialis oblique, intermedius and lateralis CSA decreased from 77.63 ± 10.6 to 70.67 ± 10.03 cm^2^ (*P *< 0.0001; Figure 1), quadriceps femoris decreased from 87.02 ± 11.50 to 80.13 ± 10.92 cm^2^ (*P *< 0.0001), and the loss of total leg lean mass was 314 ± 370 g (*P* = 0.001 Doering et al., 2022). Leg press strength decreased ∼16% following the 14‐day immobilisation (−21.5 ± 11.9 kg; *P *< 0.0001; Figure 1) and ${\dot{V}}_{O_{2}\mathrm{peak}}$ was reduced ∼3% (−1.3 ± 2.3 mL kg min^−1^; *P* = 0.0173).

**FIGURE 1:**
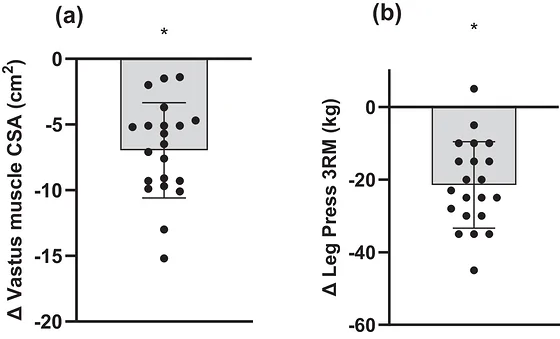
Vastus muscle group cross sectional area (CSA) (a) and leg press three repetition maximum (3RM) (b). Data are the delta change from day 0 to day 14 of knee brace limb immobilisation including mean ± standard deviation and individual response data in the young men (*n*  = 21 participants). *Data were significantly different pre‐to‐post immobilisation at *P* < 0.0001.

### 3.2 Gene expression and promoter methylation integration

The initial RNA‐seq dataset detected 14,571 genes, of which 79.0% had a matching promoter methylation signal. Genes not present in the methylation dataset (3061; 21.0%) were absent or poorly represented (<5 probes) on the EPIC (850K) array. Conversely, genes with a methylation signal (4923) but absent in the RNA‐seq dataset were either not expressed in skeletal muscle or not robustly detected at the sequencing depth used. Only genes with a corresponding ENTREZID were included in the methylation dataset (16,433 genes). This resulted in a list of 11,510 mutually identified genes. These paired datasets underwent mCSEA integration analysis, which identified negatively correlated relationships between gene expression and promoter methylation at the sample level.

Following 3 days of limb immobilisation, 308 negatively correlated genes were identified (FDR < 0.1), while at 14 days post‐immobilisation, 380 negatively correlated genes were identified. All correlation coefficients indicated at least a moderate negative correlation (*r* ≤ −0.4). Of these negatively correlated genes, 71 genes were shared between these two time points. At day 3 the distribution of correlated genes was skewed more towards upregulated/hypomethylated genes (191 genes; 62.0%) compared with downregulated/hypermethylated genes (117 genes; 38.0%). By day 14 the distribution of genes was essentially uniform, 194 upregulated/hypomethylated genes and 186 downregulated/hypermethylated genes.

The top 10 genes at day 3 and day 14 showed a moderate (>−0.6) negative correlation (Tables 1 and 2) and several were genes associated with skeletal muscle atrophy (*HDAC4*, *GADD45A* and *CHRNA1*). These three key atrogenes (*HDAC4*, *GADD45A* and *CHRNA1*) appear to be regulated by DNA methylation, specifically hypomethylation, leading to upregulation of gene expression at day 3 (Figure 2). However, further interrogation employing an expanded list of putative atrogenes showed most did not have a negative correlation between promoter methylation and gene expression (Table 3), but five were negatively correlated at day 3 and nine at day 14 (Table 2). At day 3 these included *CHRNA1*, *GADD45A* and *HDAC4*, as well as *AKT1* (translation; Figure 2) and *TRIM55* (proteolysis). At day 14 significantly correlated genes were primarily implicated in translation (*AKT1*, *EIF3F*, *EIF3K*, *EIF4EBP2*) and proteolysis (*FBXO30*, *FBXO31*, *MSTN*, *TRIM45*), while *CHRNA1* also remained significantly correlated at day 14.  Day 14 correlations showed a positive association between only *MUSK* (*r* = 0.47, *P* = 0.0322), *AGRN* (*r* = 0.45, *P* = 0.0408) and *COL13A1* (*r* = 0.56, *P* = 0.00761) with neuromuscular strength (Figure 3).

**TABLE 1:** Top 10 up‐ and downregulated, negatively correlated genes at day 3 between transcriptomic and epigenetic datasets.

Gene symbol	Correlation coefficient	FDR	Gene expression (logFC)	Methylation (NES)	No. of leading edge probes
Up					
*RAB30*	−0.710	0.000189	0.554	−1.573	14/53
*MAP3K14*	−0.709	0.000189	0.694	−1.938	9/28
*HDAC4*	−0.708	0.000189	0.977	−0.750	18/73
*GADD45A*	−0.705	0.000193	1.428	−1.493	7/10
*FAM219A*	−0.700	0.000220	0.356	−1.006	2/6
*DVL3*	−0.696	0.000224	0.254	−1.443	2/6
*MAP4*	−0.681	0.000400	0.404	−1.737	7/28
*NNAT*	−0.680	0.000400	0.935	−1.903	24/105
*CHRNA1*	−0.675	0.000442	1.505	−1.547	3/7
*ARNT*	−0.674	0.000442	0.246	−1.466	4/13
Down					
*ZNF256*	−0.437	0.0875	−0.251	0.926	7/13
*TRIB1*	−0.452	0.0718	−0.687	0.885	5/19
*SLC30A9*	−0.535	0.0172	−0.227	0.880	7/12
*PRSS23*	−0.551	0.0127	−0.496	1.270	3/15
*LOC728024*	−0.458	0.0665	−0.153	0.834	3/6
*FLYWCH2*	−0.592	0.00586	−0.500	0.717	8/19
*COMTD1*	−0.481	0.0464	−0.522	1.753	7/9
*FAM43A*	−0.486	0.0429	−0.395	1.346	12/20
*ZNF337‐AS1*	−0.472	0.0531	−0.180	1.016	3/5
*NANP*	−0.518	0.0234	−0.180	1.204	6/17

Abbreviations: FC, fold change; FDR, false discovery rate; NES, normalised enrichment score.

**TABLE 2:** Pearson's correlation for atrogenes of interest at day 3 and day 14 transcriptomic and epigenetic datasets.

Gene symbol	Day 3		Day 14		No. of leading edge probes
	Correlation	FDR	Correlation	FDR	
*AKT1*	−**0.475**	0.050	−**0.423**	0.093	11/19
*CHRNA1*	−**0.675**	0.0004	−**0.639**	0.003	3/7
*EIF3F*	−0.094	0.870	−**0.437**	0.080	6/14
*EIF3K*	−0.308	0.326	**0.513**	0.019	11/14
*EIF4A2*	−0.119	0.827	0.018	0.968	4/9
*EIF4EBP1*	0.199	0.622	−0.386	0.136	5/10
*EIF4EBP2*	0.121	0.810	−**0.436**	0.080	3/9
*FBXO21*	−0.047	0.945	0.047	0.908	6/11
*FBXO30*	−0.406	0.125	**0.538**	0.013	8/17
*FBXO31*	−0.422	0.104	**0.531**	0.014	9/43
*FBXO32*	−0.355	0.214	−0.042	0.915	4/24
*FBXO36*	−0.175	0.708	−0.064	0.866	3/11
*GADD45A*	−**0.705**	0.0002	−0.396	0.121	7/10
*HDAC4*	−**0.708**	0.0002	−0.241	0.425	18/73
*MSTN*	0.249	0.478	−**0.464**	0.057	3/5
*MYOG*	−0.035	0.957	−0.112	0.746	11/13
*TRIM28*	−0.170	0.719	0.043	0.917	9/9
*TRIM41*	−0.301	0.343	−0.005	0.990	3/17
*TRIM44*	−0.231	0.556	0.122	0.713	6/10
*TRIM45*	−0.380	0.173	−**0.448**	0.068	4/16
*TRIM55*	−**0.477**	0.049	0.004	0.993	7/10
*TRIM63*	−0.294	0.362	0.156	0.610	5/6
*TRIM68*	−0.331	0.269	−0.076	0.839	5/16
*TRIM9*	0.366	0.184	−0.410	0.108	12/22

*Note*: Bold text shows correlation value for significantly correlated genes.

Abbreviation: FDR, false discovery rate.

**FIGURE 2:**
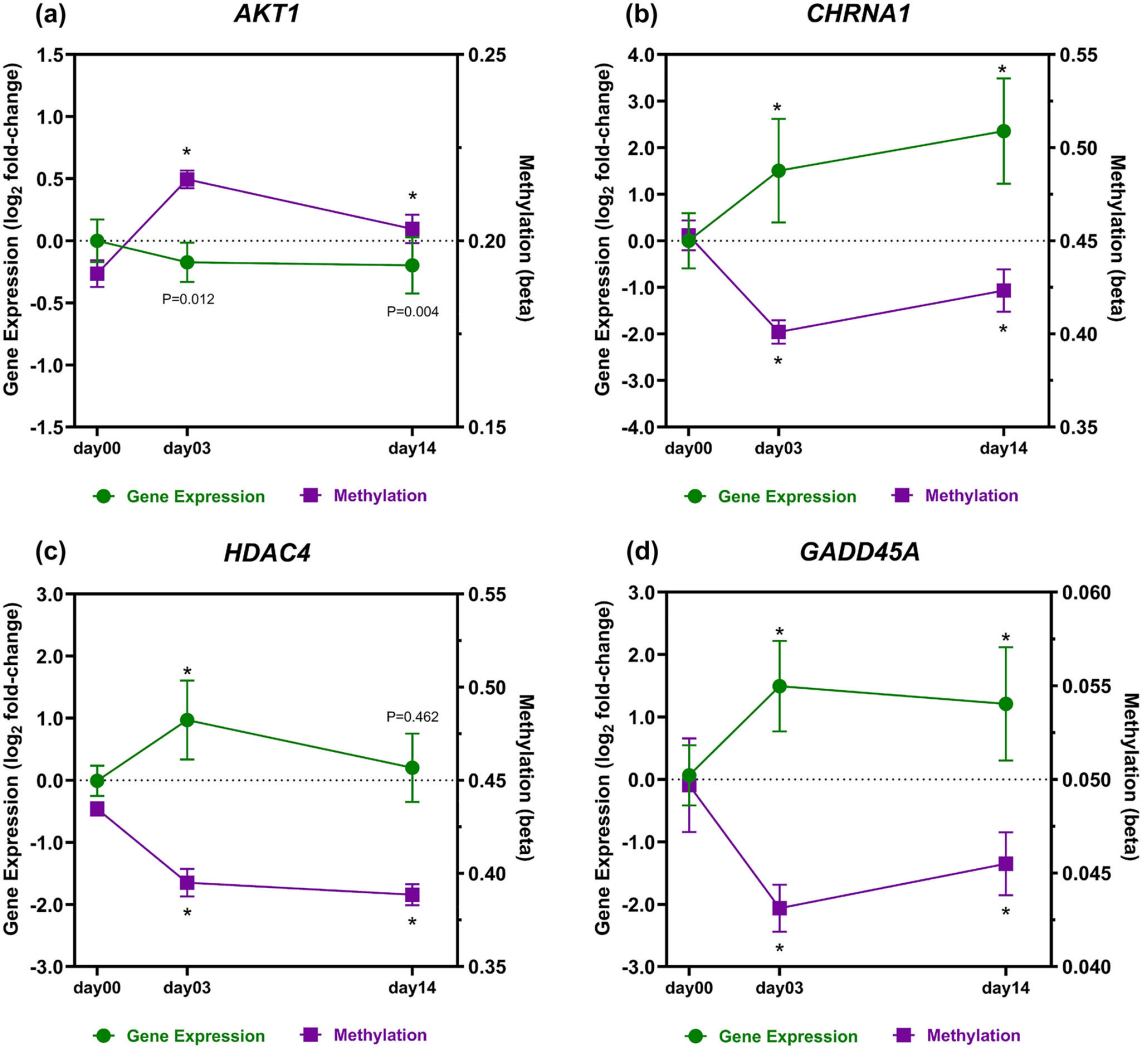
Line plot of gene expression and DNA methylation for genes of interest. Data show day 3 and day 14 of limb immobilisation normalised to day 0 for: (a) *AKT1*, (b) *CHRNA1*, (c) *HDAC4*, and (d) *GADD45A*. Green line represents gene expression, purple line represents methylation. Data shown as means ± standard deviation (*n*  = 21 participants). *P < 0.0001.

**TABLE 3:** Top 10 up‐ and downregulated, negatively correlated genes at day 14 between transcriptomic and epigenetic datasets.

Gene symbol	Correlation coefficient	FDR	Gene expression (logFC)	Methylation (NES)	No. of leading edge probes
Up					
*TXNDC6*	−0.702	0.000807	1.689	−0.856	3/14
*PDE11A*	−0.702	0.000807	1.490	−1.134	12/33
*SESN3*	−0.686	0.000842	1.010	−0.885	4/27
*SLC43A2*	−0.671	0.00137	1.623	−1.910	8/24
*CUGBP2*	−0.659	0.00178	0.539	−1.896	17/26
*CHRNA1*	−0.639	0.00293	2.357	−1.106	3/7
*PSAP*	−0.627	0.00393	0.493	−1.236	4/11
*ENOX1*	−0.620	0.00469	0.812	−0.847	12/60
*RAB11FIP3*	−0.615	0.00526	0.479	−1.354	9/21
*ARNT*	−0.611	0.00571	0.264	−0.836	4/13
Down					
*GOLPH3*	−0.687	0.000842	−0.445	1.632	4/9
*MLEC*	−0.684	0.000842	−0.645	0.960	5/15
*CHMP5*	−0.667	0.00145	−0.677	1.526	4/14
*KIF9*	−0.658	0.00178	−0.533	0.839	9/28
*MIR24‐1*	−0.654	0.00191	−0.467	0.783	5/7
*BAG1*	−0.647	0.00238	−0.677	1.518	4/12
*C12orf34*	−0.640	0.00293	−0.706	1.387	10/21
*MRPL41*	−0.630	0.00379	−0.828	1.645	3/14
*PNPLA7*	−0.630	0.00379	−0.828	1.645	3/14
*IQCH‐AS1*	−0.620	0.00469	−0.543	1.228	2/10

Abbreviations: FC, fold change; FDR, false discovery rate; NES, normalised enrichment score.

**FIGURE 3:**
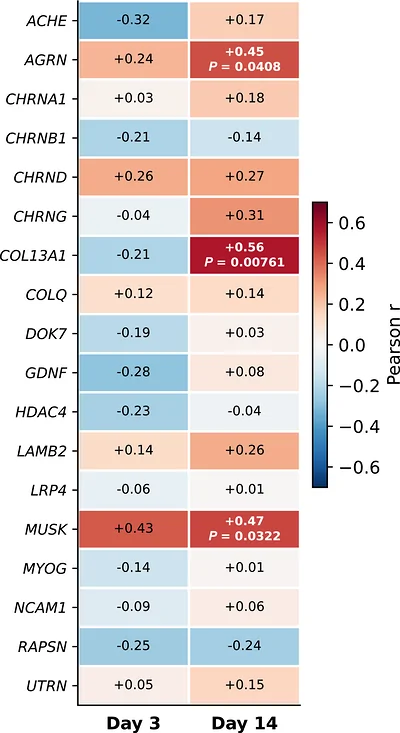
Pearson correlation coefficients between a panel of individual genes implicated in formation, maintenance or transmission at the neuromuscular junction and change in neuromuscular strength. Strength data were the delta of three repetition maximum (3RM) determined before and after 14 days of unilateral knee brace immobilisation in young men (*n* = 21 participants).

### 3.3 Over‐representation analysis

An ORA enrichment map identified 308 negatively correlated genes at day 3 (FDR < 0.1; 191 upregulated/hypomethylated and 117 downregulated/hypermethylated) and 380 negatively correlated genes at day 14 (194 upregulated/hypomethylated and 186 downregulated/hypermethylated) (Figure 4). In total there were 198 and 195 gene sets enriched (FDR < 0.1; *P* < 0.01) in the day 3 to day 0, and day 14 to day 0 comparisons, respectively. While correlated genes at day 14 were roughly evenly split between upregulation and downregulation, enriched biological process gene sets were predominantly downregulated (38 upregulated vs. 157 downregulated gene sets).

**FIGURE 4:**
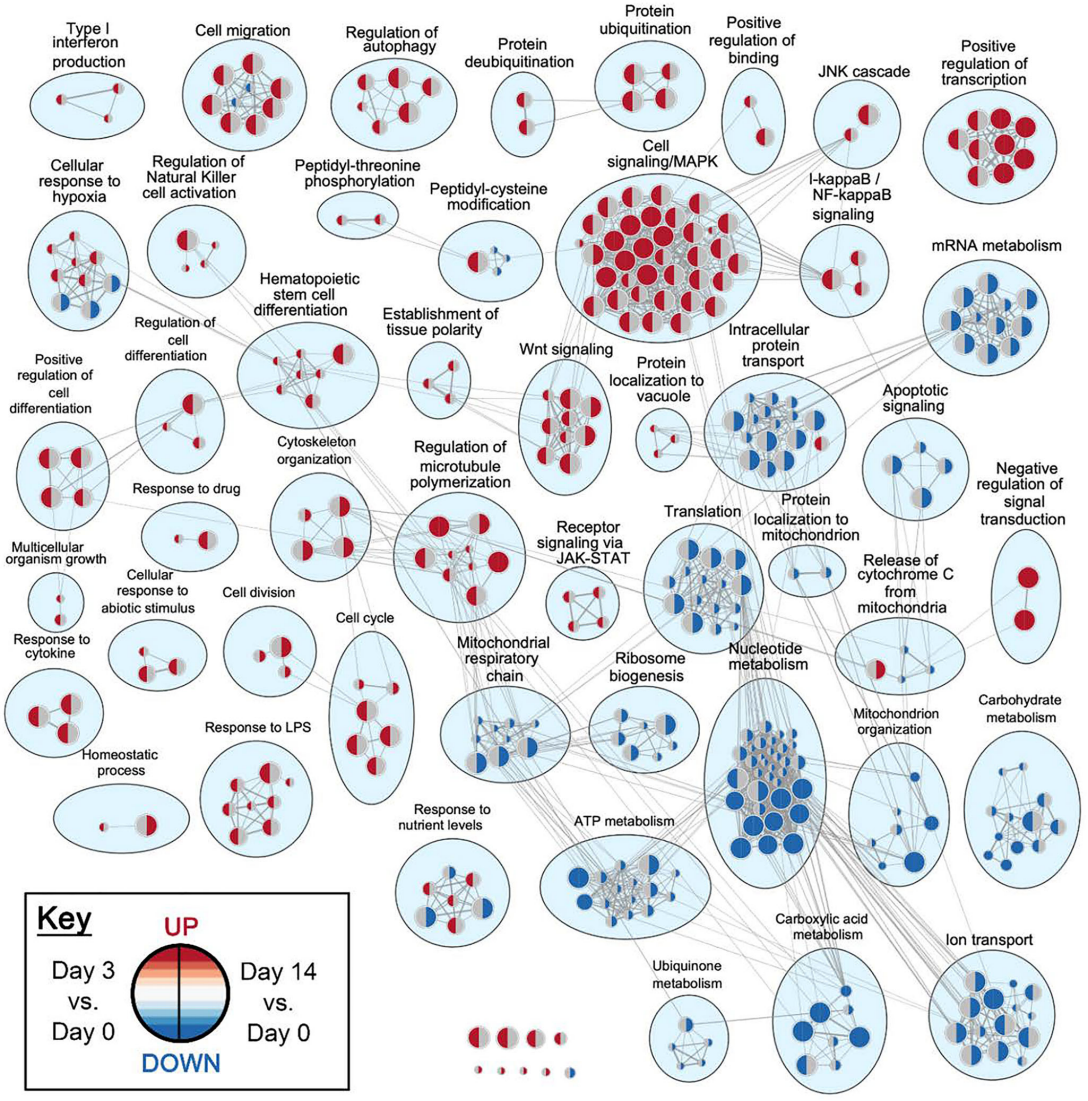
Enrichment map of GO biological processes for day 3–day 0 (left) and day 14–day 0 (right; *n* = 21 participants). Enrichment results from over‐representation analysis of GO‐BP were mapped as a network of gene sets (nodes) related by mutual overlap (edges), where red identifies upregulated/hypomethylated genes, blue identifies downregulated/hypermethylated genes, and grey identifies non‐significant gene sets following the limb immobilisation period (left: 3 days; right: 14 days). Clusters of functionally related nodes were manually labelled to summarise the included BPs. FDR < 0.1, *P* < 0.01.

To overcome gene set redundancy, BPs were grouped into larger clusters. The majority of BPs at day 3 were upregulated/hypomethylated (157 gene sets) and corresponded to cell signalling/mitogen‐activated protein kinase (MAPK) (34 gene sets), positive regulation of transcription (9 gene sets), positive regulation of microtubule polymerisation (8 gene sets), cell migration (7 upregulated gene sets, 2 downregulated gene sets), haematopoietic stem cell differentiation (7 gene sets), protein ubiquitination (4 gene sets) and the cell cycle (4 gene sets). The remainder of the 41 downregulated BPs at day 3 included nucleotide metabolism (9 gene sets), carbohydrate metabolism (10 gene sets) and carboxylic acid metabolism (5 gene sets). Upregulated BPs at day 14 corresponded to positive regulation of transcription (5 gene sets) and cell signalling/MAPK (10 gene sets).

Most GO‐BPs at day 14 were downregulated/hypermethylated and related to translation (15 gene sets), mRNA metabolism (11 gene sets), intracellular protein transport (15 gene sets), ribosome biogenesis (7 gene sets), mitochondrial respiratory chain (9 gene sets), nucleotide metabolism (33 gene sets), carboxylic acid metabolism (7 gene sets) and carbohydrate metabolism (6 gene sets). The most notable differences between RNAseq and methylation data were the exclusion of immune processes, chromatin remodelling, mRNA and rRNA processing, and angiogenesis indicating these processes may not be regulated by DNA methylation.

### 3.4 Clusters of interest

Due to the large amount of biological data generated in the present study (Figure 4), three clusters of interest were selected for further investigation using geneMANIA plots. These were chosen for their purported involvement in muscle atrophy or prominence on the enrichment map. Specifically, the clusters characterised in more detail are (i) protein ubiquitination, (ii) cell signalling, and (iii) translation.

### 3.5 Protein ubiquitination

The protein ubiquitination cluster contained four upregulated gene sets, specifically ‘protein ubiquitination’, ‘protein polyubiquitination’, ‘protein modification by small protein conjugation’ and ‘protein modification by small protein conjugation or removal’ (Figure 5). This cluster was significantly upregulated after 3 days of immobilisation and contained 27 negatively correlated genes (22 upregulated, 5 downregulated). Seven of these genes were also negatively correlated at day 14. Gene promoter hypomethylation was correlated with upregulation of two atrogenes, *HDAC4* and *TRIM55*, while hypermethylation was correlated with the downregulation in the AKT1 atrogene (Figure 1). In addition, there was upregulation/hypomethylation of proteasomal genes (*PSMD2*, *PSMD4*, *PSMC2*, *PSMC4*, *PSMC6*), which are components of the 26S proteasome involved in the degradation of ubiquitinated proteins. At day 14 this cluster was not statistically significant, which can be further evidenced by most of the methylation regulated changes in gene expression subsiding at this later time point.

**FIGURE 5:**
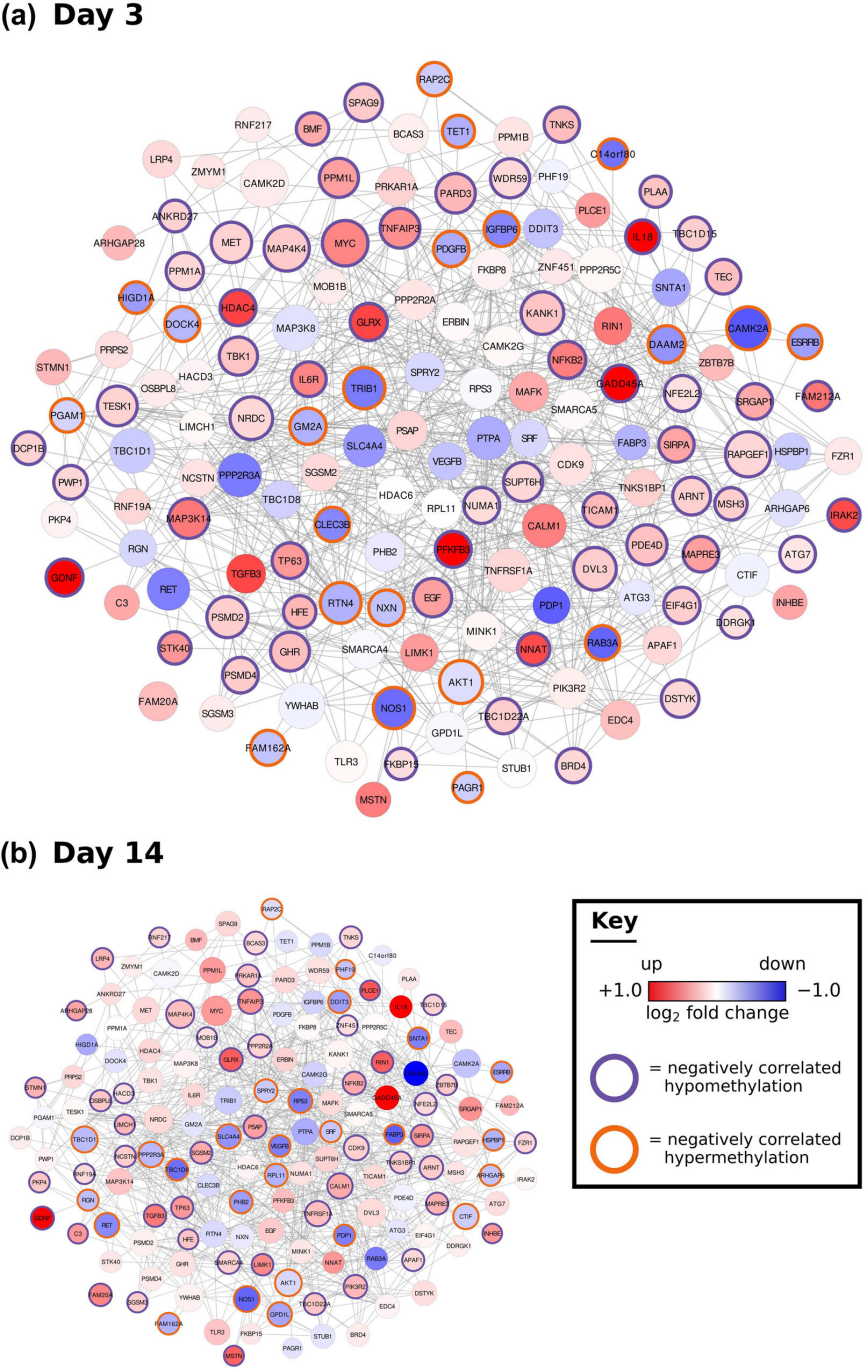
Co‐expression network of genes involved in the protein ubiquitination cluster from the enrichment map. Data shown for day 3 (a) and day 14 (b) genes involved in the corresponding cluster, as well as the top 20 co‐expressed genes (*n* = 21 participants). Node fill colour represents gene expression (log2 fold‐change; red, upregulated; blue, downregulated). The node ring represents promoter methylation that is significantly correlated with expression (purple, hypomethylation; orange, hypermethylation); nodes without a coloured ring had no significant methylation correlation. Node size is proportional to connectivity; edges represent co‐expression relationships.

### 3.6 Cell signalling/MAPK

Cell signalling was the largest cluster (36 gene sets) from the enrichment map, involving BPs such as ‘positive regulation of signalling’, ‘regulation of phosphorylation’, ‘positive regulation of intracellular signal transduction’, ‘MAPK cascade’ and ‘regulation of kinase activity’. Most BPs within this cluster were upregulated after 3 days’ immobilisation (34 gene sets), yet some continued to be upregulated after 14 days (10 gene sets), while two BPs were only upregulated after 14 days. The gene sets that were sustained at day 14 were largely related to positive regulation of phosphorylation. Three central atrogenes, *GADD45A*, *MYC* and *HDAC4*, were highlighted in this pathway as key regulators at day 3 of immobilisation (Figure 6).

**FIGURE 6:**
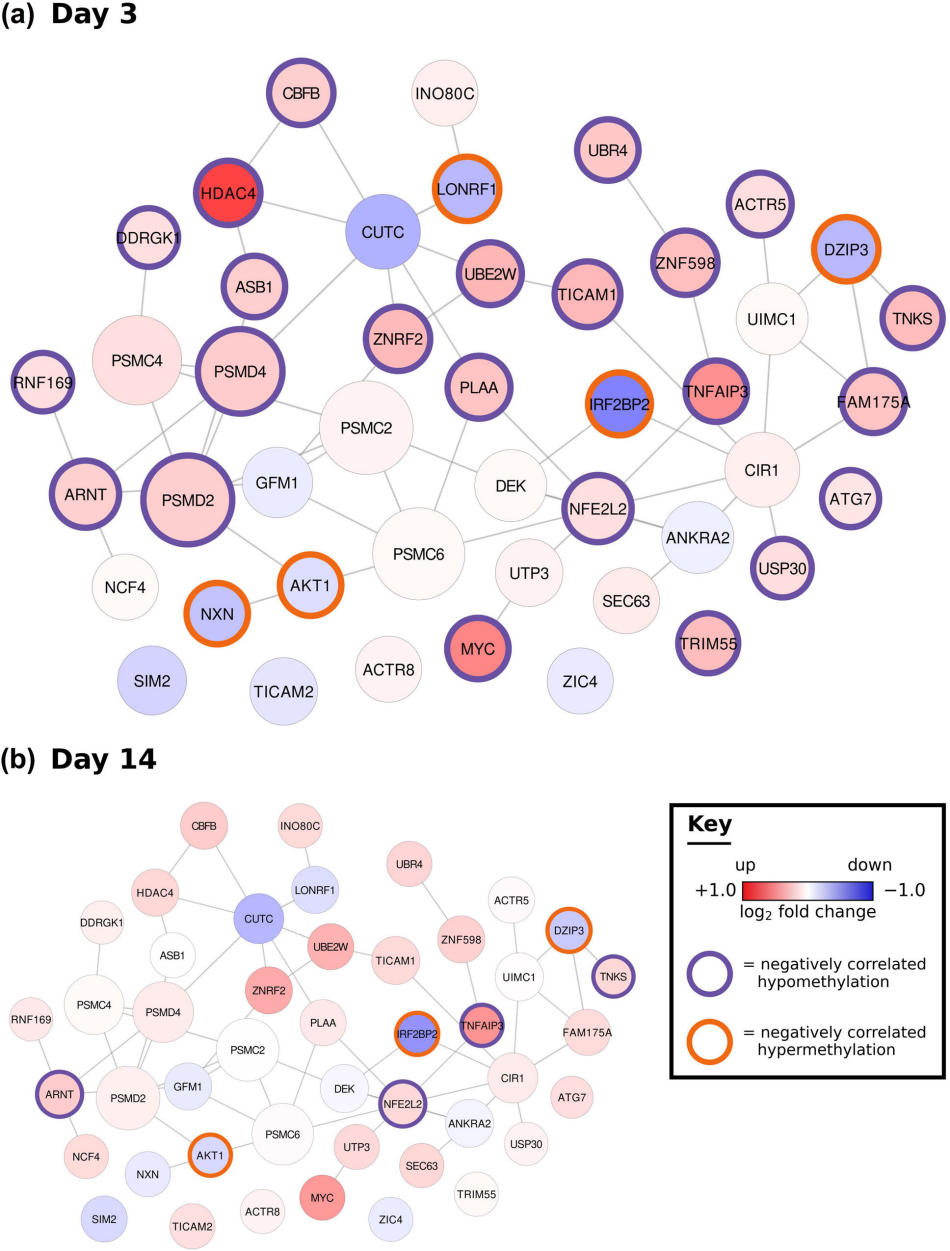
Co‐expression network of genes involved in the cell signalling/MAPK cluster from the enrichment map. Data shown for day 3 (a) and day 14 (b) genes involved in the corresponding cluster, as well as the top 20 co‐expressed genes (*n* = 21 participants). Node fill colour represents gene expression (log2 fold‐change; red, upregulated; blue, downregulated). The node ring represents promoter methylation that is significantly correlated with expression (purple, hypomethylation; orange, hypermethylation); nodes without a coloured ring had no significant methylation correlation. Node size is proportional to connectivity; edges represent co‐expression relationships.

### 3.7 Translation

Downregulation/hypermethylation of translation related gene sets was identified at day 14 (15 gene sets). There were no statistically significant BPs in this cluster at day 3, indicating changes in gene expression associated with translation as a later response to muscle unloading. Examples of BPs within this cluster include ‘translation’, ‘peptide metabolic process’, ‘mitochondrial translation’ and ‘cytoplasmic translation’. The downregulation/hypermethylation of the *AKT1* atrogene was negatively correlated at both time points (Figure 7). Translation is linked with ribosome biogenesis (7 gene sets) and mRNA metabolism (11 gene sets), which were downregulated at day 14 only. The processes in the ribosome biogenesis cluster included ‘ribosome biogenesis’, ‘ribonucleoprotein complex subunit organisation’ and ‘ribosome assembly’. Overlapping genes between translation and ribosome biogenesis included *EIF3F*, *MRPL11*, *RPL11*, *RPLP0*, *RPS19* and *RPSA*. The processes in the mRNA metabolism cluster included ‘mRNA metabolic process’, ‘nuclear‐transcribed mRNA catabolic process’ and ‘cellular nitrogen compound catabolic process’. Like ribosome biogenesis, mRNA metabolism and translation shared several ribosomal protein genes (*RPL11*, *RPL17*, *RPL18A*, *RPL39*, *RPL8*, *RPLP0*, *RPS13*, *RPS19*, *RPS3* and *RPSA*). These data show that downregulation/hypermethylation of genes associated with translation appears central to the decrease in muscle mass observed during intermediate (14 day) periods of unloading.

**FIGURE 7:**
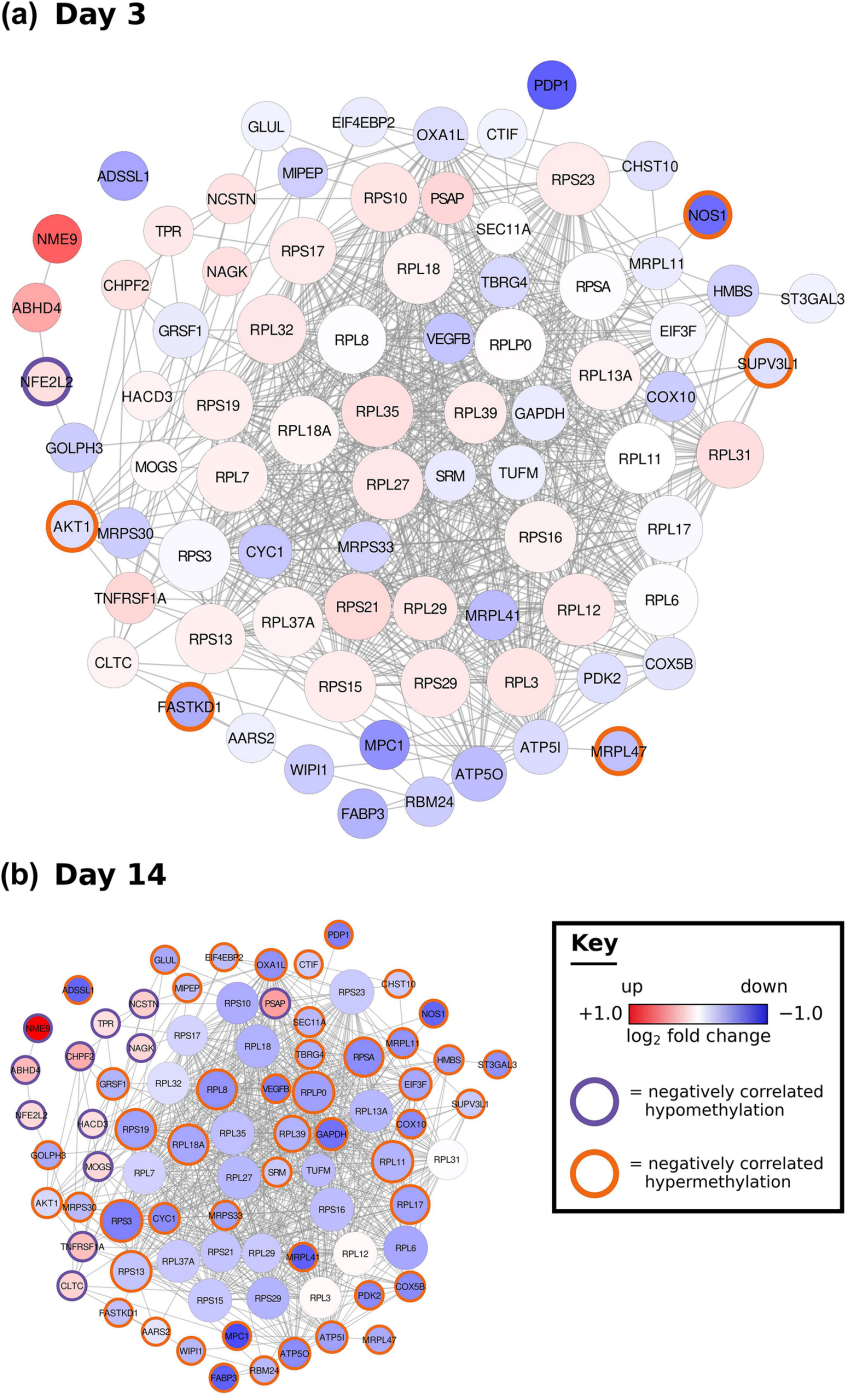
Co‐expression network of genes involved in the translation cluster from the enrichment map. Data shown for day 3 (a) and day 14 (b) genes involved in the corresponding cluster, as well as the top 20 co‐expressed genes (*n* = 21 participants). Node fill colour represents gene expression (log2 fold‐change; red, upregulated; blue, downregulated). The node ring represents promoter methylation that is significantly correlated with expression (purple, hypomethylation; orange, hypermethylation); nodes without a coloured ring had no significant methylation correlation. Node size is proportional to connectivity; edges represent co‐expression relationships.

## 4 DISCUSSION

Our multi‐omic analysis identified 71 genes with concordant methylation‐expression signatures at both day 3 and day 14 of limb immobilisation, alongside 308 and 380 genes, respectively, with significant inverse relationships between promoter methylation and gene expression. Our over‐representation analysis of these genes highlighted many important biological processes that were differentially regulated during the muscle atrophy response to unloading. These processes included translation, cell signalling within several well‐characterised pathways including MAPK, and protein ubiquitination. These novel data support the role of DNA methylation as a regulator of transcription in response to unloading and the associated decrease in muscle mass in humans.

Over‐representation analysis of enriched genes identified five key biological processes that had statistically significant corresponding upregulated/hypomethylated or downregulated/hypermethylated events at day 3 and day 14. Interestingly, at day 3 most biological processes identified were related to upregulated/hypomethylated genes (157 gene sets), compared to an increased proportion of downregulated/hypermethylated states at day 14. This biphasic response may indicate that the early upregulation of gene expression when the knee brace was first applied (0–3 days) may represent an attempt to counteract the abrupt cessation of contractile activity and maintain normal cell functions. In contrast, with prolonged unloading (day 14) widespread downregulation may reflect metabolic adaptation and a protective response to sustained net protein loss.

While there were a number of potential upregulated/hypomethylated drivers of muscle atrophy within the data, the decrease in total protein content and muscle mass of the participants could also be attributed to a decrease in the cell machinery regulating protein synthesis and translation (De Boer et al., 2007; Glover et al., 2008). Specifically, downregulation/hypermethylation of *AKT1* was negatively correlated at both time points, as well as downregulation of other notable gene families with roles in protein synthesis, that is, Ribosomal Protein S (RPS; *RPS13*, *RPS19*, *RPS3* and *RPSA*), Ribosomal Protein L (RPL; *RPL11*, *RPL17*, *RPL18A*, *RPL39* and *RPLP0*) and Eukaryotic Translation Initiation Factors (EIF; *EIF3F* and *EIF4EBP2*). These gene families are essential for the assembly of ribosomal units in preparation for translation (Yoshihama et al., 2002). Akt is a critical node in metabolic and anabolic signalling, notable for its interactions with mTOR complex 1 (mTORC1) and regulation of translation initiation (Jaiswal et al., 2022). Equally, the EIFs and ribosomal proteins represent essential cogs in the protein synthetic machinery for the assembly of new functional proteins. Consequently, the downregulation of translation initiation, translation initiation factors, and the abundance and function of ribosomes shows hypermethylation has the potential to impact multiple steps that control the protein synthesis response during muscle atrophy.

The hypermethylation of anabolic genes in our study supports previous work showing that 7–14 days after the onset of disuse, a dominant mechanism responsible for a decrease in total RNA concentration is downregulated ribosome biogenesis, characterised by a decrease in ribosomal RNA synthesis and an increase in RNA breakdown (Baehr et al., 2016; Figueir et al., 2021). Furthermore, mRNA metabolism and translation‐related biological processes in the present study shared several ribosomal protein genes (*RPL11*, *RPL17*, *RPL18A*, *RPL39* and *RPLP0*) that were part of the downregulated/hypermethylated cluster. Together, this indicates that hypermethylation of genes associated with translation may be central to the decrease in muscle mass observed during medium‐to‐long periods of unloading‐induced atrophy.

While cell signalling is heavily involved early in the response to muscle unloading, these same processes can remain active as the period of unloading or disuse is extended (Bonaldo & Sandri, 2013). Despite differences in the number of biological processes, there were a similar total number of correlated genes at each time point (83 genes at day 3, 79 genes at day 14). While this supports that many genes within signalling pathways are regulated by DNA methylation, there were more coordinated gene sets at day 3, while the patterns were more disjointed at day 14. Signalling cascades are an integral part of controlling cellular responses to changes in homeostasis, so it is unsurprising that these processes are upregulated in response to muscle unloading. The largest cluster of genes and a central part of the upregulated/hypomethylated cell signalling response was related to MAPK signalling. MAPK is involved in many cellular processes including differentiation, senescence and proliferation, and is a pathway that has previously been associated with muscle atrophy in rat models of denervation (Yuasa et al., 2018). MAPK may also upregulate *FBXO32*, one of the earliest E3 ubiquitin ligases characterised as responsive to unloading and disuse with increased mRNA expression associated with muscle atrophy (Hughes et al., 2023). The hypomethylation of MAPK signalling in response to unloading could have far‐reaching consequences for the muscle cell including its potential role in promoting muscle atrophy (Zhang & Li, 2012). Moreover, inhibition of MAPK signalling has been shown to slow muscle wasting during the early stages of atrophy in rodent unloading models (Belova et al., 2020). The substantial changes in MAPK signalling in response to a rapid halt in daily contractile overload are not unexpected given the ubiquitous expression in cells and the regulation of a multitude of cell processes by this pathway. However, it also represents a focal point for mediating appropriate anabolic versus catabolic responses to cell stress. As such, our integrated data indicate that methylation status may be an important regulatory step for the expression of signalling proteins that control muscle atrophy.

The role of protein ubiquitination in various biological processes continues to be elucidated, although the capacity of so‐called atrogenes and other E3 ligases to act as a marker of protein degradation remains contentious (Hughes et al., 2023). Key genes identified as statistically significant within the ubiquitination biological process included *HDAC4*, *TRIM55* and *AKT1*. Upregulation of *HDAC4* activates atrophy‐associated signalling pathways (Bongers et al., 2013; Choi et al., 2012), whilst *TRIM55* upregulation likely promotes protein breakdown via the ubiquitin–proteasome system (Centner et al., 2001). Downregulation of *AKT1* is associated with proteolytic activation (Sandri et al., 2004), and additional atrogenes *GADD45A* and *CHRNA1* were also implicated in the atrophy response.

The integration of the two time points allowed us to distinguish relationships that were transient from those that emerged later or persisted throughout unloading. Of the negatively correlated genes, 71 were shared between day 3 and day 14, while the remainder were time point‐specific (237 at day 3 only; 309 at day 14 only). The protein ubiquitination cluster exemplified an acute response; it was significantly enriched at day 3, but was no longer significant by day 14, with only seven of an original 27 genes retaining a significant negative correlation. This indicates that methylation‐association regulation of the early degradative response is largely transient. In contrast, translation (15 gene sets), ribosome biogenesis (7 gene sets) and mRNA metabolism (11 gene sets) reached significance only at day 14, consistent with suppression of the protein‐synthetic machinery as a later, intermediate response to unloading. *AKT1* represented a persistent relationship, being negatively correlated at both time points and linking the early signalling response to the later decline in translational capacity. Together, this temporal partitioning suggests that DNA methylation contributes to a coordinated shift from early degradation toward a later suppression of protein synthesis as immobilisation progresses.

Our data for *CHRNA1* and *HDAC4* in human muscle are consistent with findings reported by Fisher and co‐workers using denervation‐induced atrophy in rats (Fisher et al., 2017). The *CHRNA1* gene encodes a subunit of acetylcholine regulated channel gating and due to its role in propagating muscle action potentials a change in expression might have been expected with nerve silencing. We extend this finding by demonstrating a comparable response for *CHRNA1* hypomethylation and increased gene expression at day 3 and 14 of limb immobilisation. Data for *HDAC4* were also consistent with Fisher et al. (2017) given the decreased methylation and upregulated expression in our human study. *HDAC4* regulates three non‐histone protein targets (myosin heavy chain, peroxisome proliferator‐activated receptor γ coactivator 1‐α (PGC‐1α), and heat shock cognate 71 kDa protein (HSC70)) and is involved in chromatin remodelling (Drazic et al., 2016; Luo et al., 2019). Moreover, *HDAC4* has been purported to control skeletal muscle homeostasis through regulation of myosin heavy chains and the master regulator of the mitochondria, PGC‐1α (Luo et al., 2019; Olesen et al., 2010). Notably, human models of atrophy where the time course of sampling is limited can make it difficult to ascertain whether molecular changes in the cell are responsible for net protein degradation or are merely a response to muscle unloading. However, our *GADD45A* data support previous work of Bongers et al. (2013) identifying *GADD45A* as a downstream mediator of *HDAC4* that is required for *HDAC4*‐induced muscle atrophy in response to denervation in mice. Therefore, the dual *HDAC4*–*GADD45A* upregulation in the present study directly implicates this pathway in promoting unloading‐mediated loss of muscle mass and demonstrates that these genes are subject to methylation‐based regulation in human skeletal muscle. Given the agreement between these previous small animal studies and our human work, several candidate genes emerge that may be important mediators of net muscle protein degradation, and the effects were apparent across denervation and immobilisation atrophy‐inducing stimuli. Importantly, the findings of the present study provide evidence to support hypomethylation as a key regulatory process in the control of gene expression of ‘atrogenes’ in human skeletal muscle.

Previous studies have also considered the impact of unloading on skeletal muscle motor unit properties and function, not merely muscle mass (Sarto et al., 2025, 2022). Consequently, we undertook correlation analysis between the expression of primary candidate genes linked with regulation of the neuromuscular junction and the loss of leg strength that identified *COL13A1*, *AGRN* and *MUSK* genes were associated with decreased muscle function. There is a paucity of data on *COL13A1*, which is expressed in skeletal muscle near the neuromuscular junction (Latvanlehto et al., 2010), while recent findings on agrin (AGRN) indicate it is an upstream regulator of muscle‐specific kinase (MUSK) signalling that may mediate acetylcholine receptor clustering (Makanae et al., 2025). Moreover, *AGRN* and *MUSK* gene and protein expression are responsive to the type of muscle contraction undertaken during resistance training (Makanae et al., 2025). Our correlation data provide further characterisation of AGRN–MUSK showing a potential role in regulating neuromuscular junction integrity and functional strength. Together with *CHRNA1*, *HDAC4* and *GADD45A*, these emerging genes implicated in regulating neuromuscular junction integrity and function may be an important area of future enquiry in the aetiology of muscle atrophy.

Overall, our integration of promoter DNA methylation and gene expression data further supports the standalone RNAseq findings. Although somewhat expected, the data provide strong evidence for a regulatory role of promoter methylation on key genes that are involved in cell signalling (MAPK), regulating protein degradation (protein ubiquitination) and protein synthesis (translation) in human skeletal muscle. The data highlight the limitations of assessing DNA methylation changes in isolation, without supporting gene expression analysis, due to the minimal overlap in GO‐BPs in the present study. However, there were also genes that did not appear to be regulated by methylation. Accordingly, it seems logical that other regulatory mechanisms (e.g., miRNA, alternative splicing) would also regulate the atrophy response for genes not controlled by DNA methylation. Further work to elucidate these alternative processes may be useful in identifying novel regulators of muscle atrophy. In addition, we cannot rule out some aspects of heterogeneity in the atrophy response generated by a preceding exercise‐induced methylation programme in the present study. This has been termed an ‘epigenetic memory’ in skeletal muscle where hypomethylation to previous exercise stimuli may be retained during detraining and subsequently promotes an enhanced response with return to exercise (Seaborne et al., 2018; Sharples et al., 2016). Any impact of prior exercise may also be complicated by the training mode and history where resistance and endurance training, and training status, demonstrate different methylation signatures in skeletal muscle (Geiger et al., 2024); hence, our highly controlled study design that included endurance‐ and resistance‐type training and matched exercise prescription that was undertaken at the same relative load for all participants to equalise the stimulus prior to immobilisation. Whether sedentary versus exercise trained muscle promotes more or less heterogeneity in methylation response and gene expression is an interesting question for future work (Etayo‐Urtasun et al., 2024). Nonetheless, prior exercise training could have a potential therapeutic effect, at least on the methylome, that might promote recovery after immobilisation. Accordingly, our findings may be a reference data set from optimal pre‐atrophy adapted muscle with which to compare future studies of unloading, disuse or pathological muscle wasting responses.

### 4.1 Conclusion

This study undertook the integration of transcriptome and methylome data and identified key genes and regulatory pathways associated with knee‐brace immobilisation induced skeletal muscle atrophy in humans similar to the immobilisation following injury. A clear understanding of the regulatory role of DNA methylation in many adaptive and maladaptive responses in skeletal muscle remains elusive, due to the complex nature of methylation patterns in promoter regions, gene bodies and CpG islands, and the variable number of methylation sites per gene region. Nonetheless, we were able to characterise changes in gene expression related to hypo‐ and hyper‐methylation during muscle unloading. Determining whether attenuated synthesis or exacerbated degradation is primarily driving atrophy may be a continuing scientific debate but is arguably less important than identifying key molecular targets for interventions to mitigate the debilitating impacts of muscle wasting. Importantly, our data provide support for a role of methylation in control of gene expression that downregulates processes for protein synthesis and upregulates those that promote protein degradation in skeletal muscle. Consequently, this integrated analysis offers novel insight into biological processes controlling muscle atrophy and into associations at the individual gene level that may be useful in developing strategies for reducing loss of muscle mass with injury or as a secondary complication in chronic disease.

## AUTHOR CONTRIBUTIONS

Conception and design: Vernon G. Coffey, Kevin J. Ashton. Acquisition, analysis or interpretation of data: Jamie‐Lee M. Thompson, Thomas M. Doering, Kristen L. Mackenzie‐Shalders, Boris P. Budiono, Kevin J. Ashton, Paul J. Dunn, Vernon G. Coffey. Drafting the work or revising it critically: Jamie‐Lee M. Thompson, Thomas M. Doering, Kristen L. Mackenzie‐Shalders, Boris P. Budiono, Kevin J. Ashton, Paul J. Dunn, Vernon G. Coffey. All authors have read and approved the final version of this manuscript and agree to be accountable for all aspects of the work in ensuring that questions related to the accuracy or integrity of any part of the work are appropriately investigated and resolved. All persons designated as authors qualify for authorship, and all those who qualify for authorship are listed.

## CONFLICT OF INTEREST

None declared.

## GENERATIVE AI STATEMENT

There was no use of GAI in the present study.

## Data Availability

All sequencing data that support the findings of this study have been deposited in the NIH NCBI Sequence Read Archive (SRA) under the accession number PRJNA1462955 available via PRJNA1462955 – SRA—NCBI. The methylation array dataset has been submitted to the NCBI Gene expression omnibus (GEO) database under the tracking number GSE330512 and can be accessed via the NCBI databases (GEO Accession viewer—GSE330512).
